# Transcutaneous auricular vagus nerve stimulation during a 3-back task: a high-density functional near-infrared spectroscopy study

**DOI:** 10.1007/s00221-026-07354-2

**Published:** 2026-07-12

**Authors:** Michal Holowacz, Caitlin Illingworth, Jasmine Swalwell, Daniel Blackburn, Ali Ali, Arshad Majid, Li Su, Sheharyar S. Baig

**Affiliations:** 1 Sheffield Institute for Translational Neuroscience, School of Medicine and Population Health, University of Sheffield, Sheffield, UK; 2 Department of Psychiatry, School of Clinical Medicine, University of Cambridge, Cambridge, UK

**Keywords:** Vagus nerve stimulation, Functional near-infrared spectroscopy, Non-invasive brain stimulation, Cognitive impairment, Stroke

## Abstract

Working memory issues are an often life-impairing deficit seen in several neurological disorders. Transcutaneous vagus nerve stimulation (tVNS) is a promising neuromodulatory adjunct to cognitive training and may improve working memory. High-density functional near-infrared spectroscopy (HD-fNIRS) is a modern neuroimaging modality that can assess cerebral blood flow in cortical areas. In the current study, we used HD-fNIRS to determine the effects of tVNS during a working memory task. Twenty-two healthy adult participants (12 female, 10 male) performed a 3-back task in a block design whilst high-density fNIRS was recorded across the bilateral prefrontal cortex using 36 sources and 48 detectors (1728 channels). Sham (earlobe) tVNS was delivered during the first task trial and active (tragus) tVNS was delivered during the second task trial. Statistical analysis was performed at a group level within trial (task - baseline) and between trials (active tVNS vs. sham tVNS) using node-level and regional approaches. Task-related increases in HbO were seen in the right dorsolateral middle frontal gyri, left inferior frontal gyrus and right lateral orbitofrontal cortex under both active and sham tVNS. Decreases were observed in the bilateral superior medial frontal gyri and medial orbital frontal cortices. No significant differences were seen between sham and active tVNS. Simultaneous recording of HD-fNIRS during a 3-back task and concurrent tVNS was feasible and well-tolerated. Robust task-related activation was seen in lateral frontal areas. No significant active-versus-sham differences in cortical activity or behavioural performance were observed under the stimulation parameters used here; however, this finding should be interpreted cautiously because the fixed-order design may have introduced fatigue or habituation effects. HD-fNIRS could be used in future studies of working memory and neuromodulation in clinical cohorts.

## Introduction

Working memory is described as the ability to store and manipulate information for a short period of time (Baddeley 1992). It is necessary to support goal-directed behaviour and decision-making (Baddeley 2010). In neurological disorders such as mild cognitive impairment (MCI), working memory is impaired (Saunders and Summers 2009; Jongsiriyanyong and Limpawattana 2018) and is associated with reduced quality of life (Ruan et al. 2014). Non-invasive or transcutaneous vagus nerve stimulation (tVNS) is a promising neuromodulation technique in cognitive impairment (Wang et al. 2024). Afferent tVNS modulates cholinergic, noradrenergic and serotonergic signalling via brainstem nuclei (Colzato and Beste 2020; Shiozawa et al. 2014). It has previously been shown to improve working memory in healthy young adults (Sun et al. 2021) and to support motor learning and language learning in other neurological disorders (Baig et al. 2021; Murphy et al. 2022; Austelle et al. 2024). A previous double-blind randomised clinical trial reported that 24 weeks of tVNS treatment is feasible in MCI (Wang et al. 2022).

One of the challenges of monitoring the effects of tVNS and determining whether there is an individual-level response is the lack of robust biomarkers of vagus nerve activation during cognitive tasks (Burger et al. 2020). Whilst functional magnetic resonance imaging (fMRI) demonstrates that tVNS activates brainstem and cortical projections of the vagus nerve (Borgmann et al. 2021), performing concurrent cognitive tasks alongside non-invasive brain stimulation is challenging due to the incompatibility of electronic devices with the magnetic field of a scanner (Badran et al. 2017).

Functional near-infrared spectroscopy (fNIRS) uses near-infrared light to determine cerebral blood flow by measuring changes in the concentration of oxygenated haemoglobin (HbO) and deoxygenated haemoglobin (HbR) (Yücel et al. 2021). It can be performed in more ecologically valid settings (Pinti et al. 2015) and alongside non-invasive brain stimulation (Curtin et al. 2019). High density fNIRS (HD-fNIRS) or high density diffuse optical tomography (HD-DOT) refers to arrays of dense, overlapping sources and detectors at multiple separation distances that enables portable functional neuroimaging with similar spatial resolution to fMRI (Vidal-Rosas et al. 2023; Wheelock et al. 2019).

No previous studies have determined whether tVNS can modulate cortical activation during a working memory task. This single-blind pilot study aimed to assess whether auricular (tragus) tVNS could (a) improve performance in a N-Back task and (b) change prefrontal cortical activity as assessed via HD-fNIRS. We hypothesise that conventionally used stimulation settings will modulate task-related cortical activity during the 3-back task in healthy volunteers, reflected in changes in both the magnitude and spatial distribution of oxygenated haemoglobin responses.

## Materials and methods

### Participants

A total of 24 healthy adults (13 female, 11 male) were recruited through convenience sampling from staff and students at the University of Sheffield. Participants were excluded if they had contraindications to use of HD-fNIRS (open wound on scalp/head) or tVNS (pregnancy, prior vagotomy, non-removable piercings at left tragus or earlobe, implanted electronic medical devices, known symptomatic bradycardia or conductive heart block, or carotid artery stenosis exceeding 50%).

### Experimental design

A within-subjects block design was employed to investigate the cortical effects of tVNS on cognitive tasks. Written informed consent was obtained from all participants. Demographic information was collected, and participants were checked for contraindications to tVNS. Each participant completed a semantic verbal fluency task and a 3-back N-Back task under sham and then active tVNS. The N-Back task results are the focus of the current study. Data collection took place in a quiet room at the University of Sheffield or in the Royal Hallamshire Hospital Biomedical Research Centre.

### N-Back task

Stimulus presentation and response logging were controlled with PsychoPy v2024.2.24 running under Python 3.11 on a 60 Hz laptop display (1920 × 1080 px). The experimental sequence consisted of alternating four 0-back and three 3-back blocks. A custom script was coded for this study; it generated digit stimuli (2–9, white on black) for 1.5 s, separated by a 0.5 s blank screen. The 3-back task blocks lasted 60 s and required participants to press the spacebar on a keyboard when the number currently on the screen matched the number seen three steps back in the sequence.

**Fig. 1:**
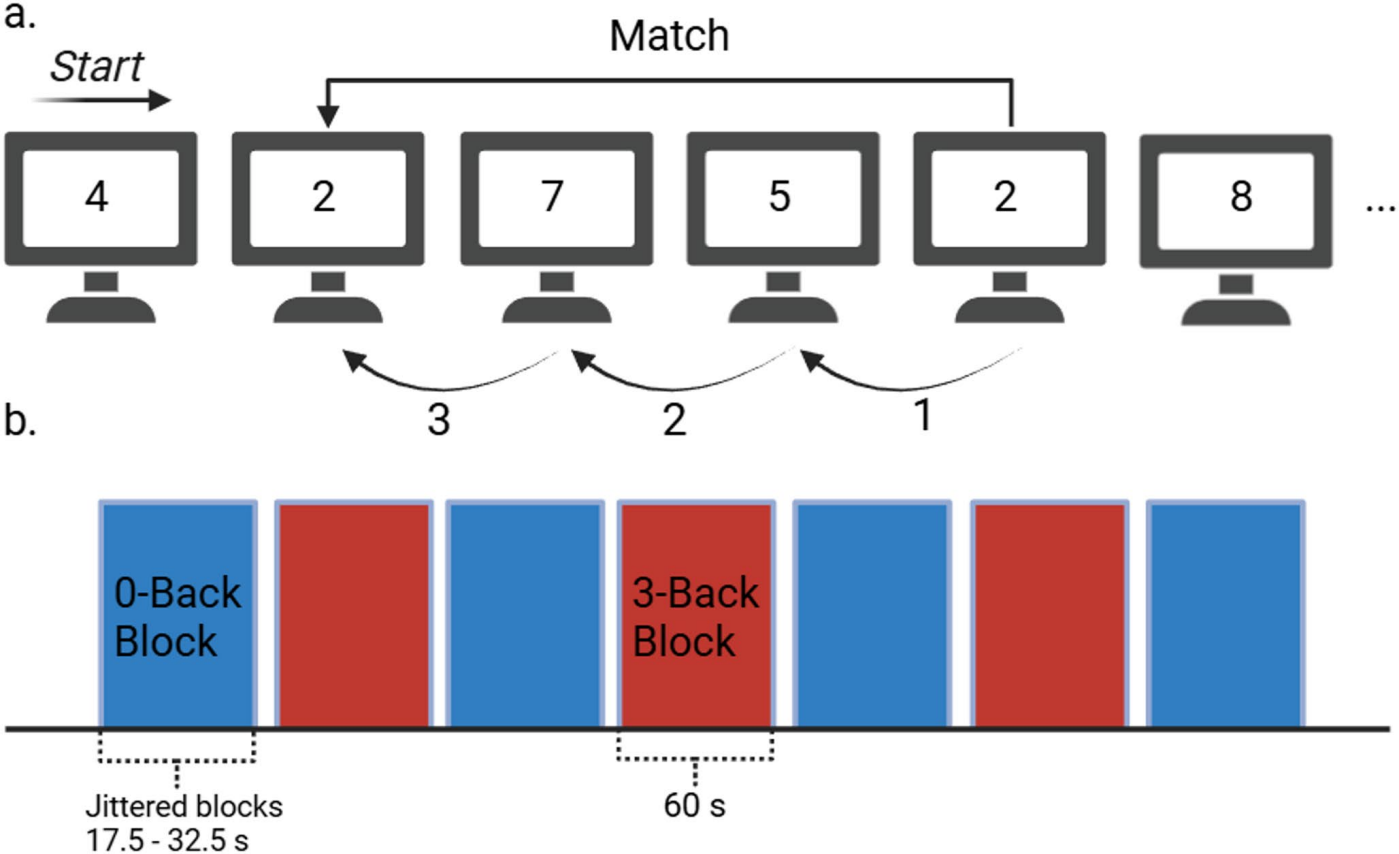
**a** A schematic of the 3-back task. **b** An overview of the block design

A schematic representation of the numeric 3-back sequence is shown in Fig. 1a. The block design is illustrated in Fig. 1b. The 0-back task was chosen as the baseline (rest) interval to match the 3-back task for visual input, motor demand of button pressing and general attention so that the comparison was of the working memory load of the task. The 0-back blocks lasted 17.5–22.5 s (jittered) for the first 9 participants but changed to 27.5–32.5 s for the remaining participants (*n* = 13) to allow more time for the haemodynamic response to return to baseline. Participants were given a practice round prior to recording and given the opportunity to ask questions.

For accuracy and reaction time during the 3-back task, the scores were averaged across the 3 blocks within each stimulation condition and participant. Accuracy was calculated as the proportion of target trials with a correct button press within the response window.

### HD-fNIRS acquisition

**Fig. 2:**
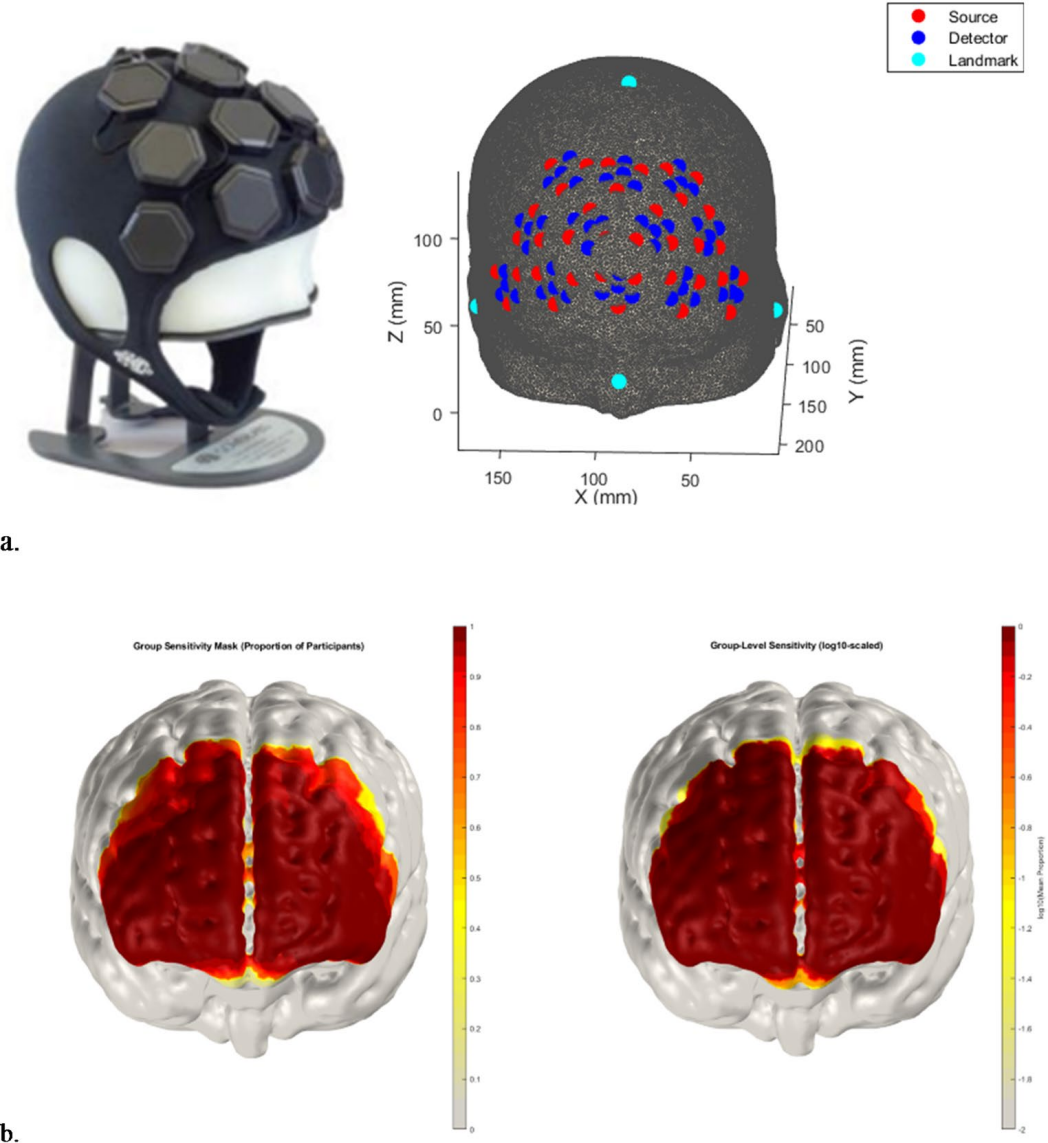
**a** Source-Detector Registration to template brain showing locations of optodes. **b** Sensitivity mask for HD-fNIRS illustrating average sensitivity across all participants. Node sensitivity is defined as using Uchitel (2022) thresholds for detecting changes in HbO and HbR during the parcellation step

A high-density continuous wave fNIRS system (LUMO; Gowerlabs Ltd., UK) with 12 tiles placed in a bilateral prefrontal cortex array was used to collect fNIRS data (Fig. 2). The LUMO system consists of multi-distance overlapping channels, enabling the dissociation of haemodynamic data from the scalp (10–12 mm channels) and the cortex (12–42.5 mm). Each tile contains three dual-wavelength LED sources (735|850 nm) and four photodiode detectors, for a total of 36 sources and 48 detectors, where each source forms a channel with each detector for a total possible 1728 channels. Of these, approximately 464 channels fall within the range used for analysis (10–42.5 mm).

### tVNS delivery

**Fig. 3:**
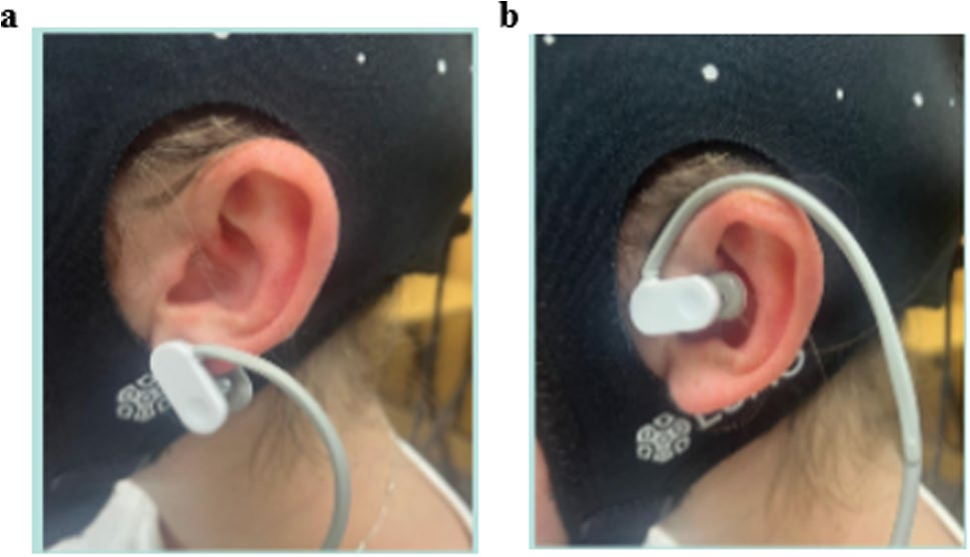
Stimulation sites for sham (**a**) and active tVNS (**b**)

tVNS was delivered using the Nurosym device (Nurosym Ltd., United Kingdom) at 25 Hz with a 250-µs pulse width. Stimulation began shortly before the task and continued through the rest interval. Active stimulation was applied to the left tragus, whereas sham stimulation was applied to the left earlobe (see Fig. 3), which lacks vagal innervation (Peuker and Filler 2002). Stimulation intensity was set at the midpoint between each participant’s perceptual and pain thresholds to ensure matched subjective intensity across conditions.

### Behavioural analysis

To investigate the potential effect of taVNS on cognitive performance, the accuracy for each participant during the 3-back trials was calculated from the number of correct responses/ the total number of positive trials. Mean reaction time was calculated for all the correct trials (ms). Paired t-tests were conducted to analyse the change in accuracy and reaction time between each condition. To assess the interpretability of non-significant behavioural findings, post hoc sensitivity analyses were conducted for the final behavioural sample sizes using a two-tailed paired-samples t-test, α = 0.05, and 80% power.

An exploratory Spearman correlation was conducted between active-minus-sham changes in task-evoked **Δ**HbO within the right dorsolateral middle frontal gyrus and active-minus-sham changes in 3-back accuracy.

### fNIRS preprocessing

Preprocessing was carried out in MATLAB R2023b (MathWorks, Natick, MA) using a custom workflow that integrated functions from Homer2 (Huppert et al. 2009) and the DOT-HUB toolbox (https://github.com/DOT-HUB/DOT-HUB*).*

Raw light-intensity data were first transformed into changes in optical density (ΔOD) via Homer2. Channels were discarded if raw intensity fell outside the range [$0\,\mathrm{to}\,1 \times 10^{11}$], if the signal-to-noise ratio was below 12, or if source-detector separation exceeded 100 mm (Uchitel et al. 2022). Motion artefacts were identified using amplitude and standard deviation criteria (0.1 and 50, respectively) and corrected using HmrMotionCorrSplineSG, which applies spline interpolation (spline parameters = 0.99) combined with Savitzky-Golay filtering (10-s window) (Jahani et al. 2018).

ΔOD signals were then converted into HbO and HbR concentration changes using hmrOD2Conc with a differential pathlength factor of 6. To reduce superficial contamination, short-channel regression (0–12 mm) was applied. The resulting signals were filtered using a third-order Butterworth band-pass filter (0.01–0.1 Hz). Task-locked haemodynamic responses were extracted using block averaging time-locked to stimulus onset, with − 2 to 0 s serving as the baseline window and 10–20 s representing the task-evoked response period.

### Image reconstruction and parcellation

For each participant, cortical oxygenation changes were constructed using a tetrahedral grey-matter mesh derived from the MNI-152 templated head model (Maintz and Viergever 1998). Concentration changes were first converted to optical density using DOTHUB_hmrConc2OD. Light-propagation modelling was performed using DOTHUB_make_Jacobian to generate the forward model, after which a zeroth-order Tikhonov-regularised inversion (regularisation parameter = 0.01) was applied to obtain volumetric reconstructions.

### Node-level statistical analysis

Participants were excluded from further analysis if < 1/3 of channels within 10–42.5 mm met pruning criteria (Fiske et al. 2022). The average HbO concentration for each reconstructed node across all participants at baseline (-2–0 s) and task (10–20 s) was extracted for each condition. For within-condition analysis paired t-tests were conducted for each node to compare node-level changes in HbO during the task versus baseline periods. For between condition analysis, the mean change in HbO concentration from baseline to task was calculated for each condition and used to compare between conditions. The derived t-stats were then plotted on the cortical reconstruction, with only nodes with *p* < .05 plotted. To control for multiple comparisons, an FDR correction was performed.

### Reconstruction parcellation

**Fig. 4:**
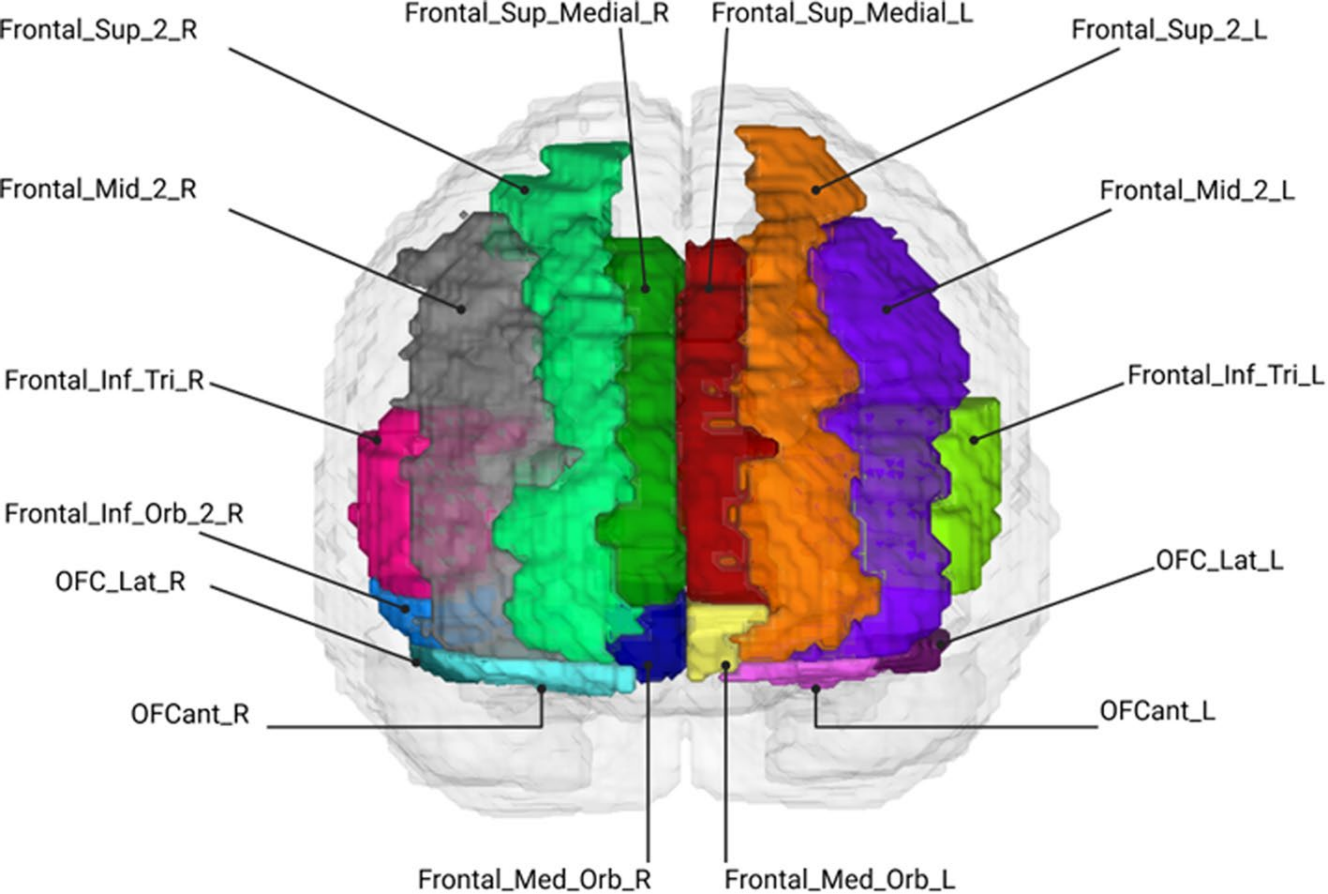
Cortical regions from the AAL2 atlas targeted in this study using the LUMO HD-fNIRS cap. These parcels defined the 15 frontal ROIs for group-level parcellation analysis

Reconstructed data for each participant were then parcellated using the AAL2 atlas (Rolls et al. 2015), assigning each grey-matter mesh node to one of 15 frontal regions of interest (Fig. 4). Parcels were retained only when more than 50% of their constituent nodes were classified as sensitive (if its sensitivity in the Jacobian matrix exceeded 5% of the maximum value of the normalised Jacobian for both HbO and HbR wavelengths) (Butters et al. 2025; Uchitel et al. 2022). A single time-course per parcel was created by averaging the time series across all sensitive nodes belonging to that parcel.

### Parcellation statistical analysis

**Fig. 5:**
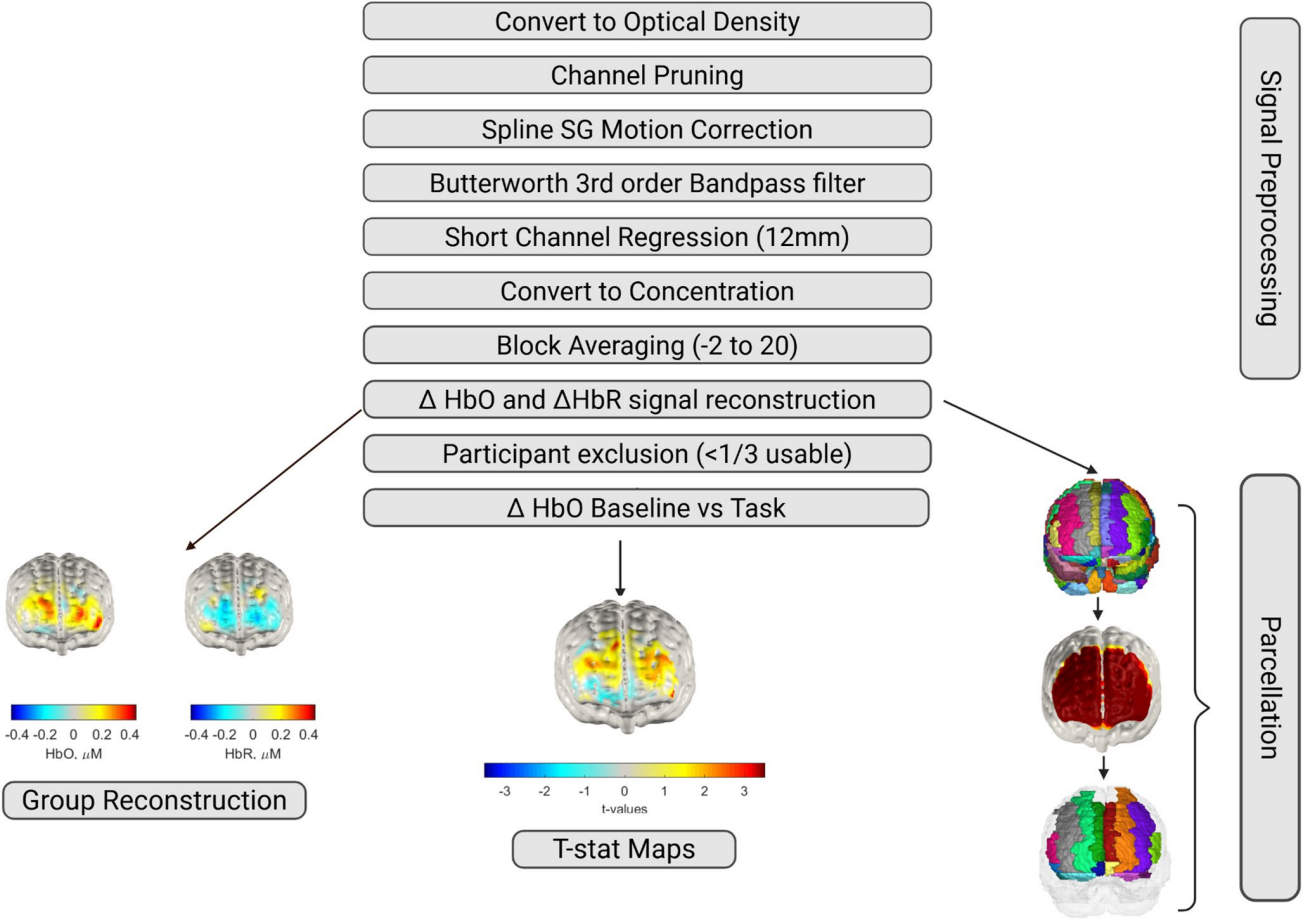
Overview of the data analysis pipeline

For each parcel, task-evoked changes in HbO and HbR were calculated based on baseline and task windows. Only Δ*HbO* was analysed in the present study due to its higher signal-to-noise ratio (Tachtsidis and Scholkmann 2016). Mean HbO concentrations were extracted for each participant, parcel, and condition, providing the inputs for group-level statistical analyses. Paired *t*-tests were conducted for all parcels between the group average baseline and task window active mean HbO. Multiple comparisons were corrected using the false discovery rate (FDR) method. Both corrected and uncorrected *p*-values are reported. An overview of the whole analysis pipeline is shown in Fig. 5.

## Results

### Participant characteristics

Participants were recruited from April to July 2025. Due to an experimental stimuli error, two participants did not complete the N-back task, leaving a cohort of 22 healthy adults (12 female, 10 male). Participants were excluded from further analysis if < 1/3 of channels within 10–42.5 mm met pruning criteria (Fiske et al. 2022). No participants were excluded from the node-level analysis. The characteristics for each cohort used are reported in Table 1.

**Table 1:** Participant characteristics of the final analysed cohort

Characteristic	Total cohort
	*n* (%)
n	22
Age (years)	25.0 (5.9)
Sex	
Female	12 (54.5%)
Male	10 (45.5%)
Race/ethnicity	
White	19 (83.3%)
South Asian	1 (4.5%)
Chinese	1 (4.5%)
Mixed Race: White and Black	1 (4.5%)
First language	
English	18 (81.8%)
Mandarin	1 (4.5%)
French	1 (4.5%)
Spanish	1 (4.5%)
Polish	1 (4.5%)
Hair colour	
Brown	17 (77.3%)
Blonde	2 (9.1%)
Black	1 (4.5%)
Red	1 (4.5%)
No hair	1 (4.5%)
Handedness	
Right	19 (86.4%)
Left	3 (13.6%)

Values are presented as mean (SD) for continuous variables and n (%) for categorical variables

### tVNS parameters

There was no significant difference in the mean (SD) stimulation amplitude for active (tragus) and sham (earlobe) stimulation conditions (*t* (41) = 1.85, *p* = .07). Mean stimulation amplitudes are shown in Table 2. tVNS was well tolerated with no significant reported adverse events.

**Table 2:** Stimulation amplitudes for sham and active tVNS conditions

Condition	Perception threshold (mA)	Pain threshold (mA)	Final threshold (mA)
Sham	16.0 (3.2)	22.0 (4.4)	18.5 (4.2)
Active	17.4 (4.6)	22.8 (4.1)	19.4 (4.1)

Values are presented as mean (SD) in milliamperes (mA). Perception threshold refers to the minimum current at which stimulation was detected by the participant. Pain threshold refers to the maximum tolerable current. Final threshold corresponds to the stimulation intensity applied during the task

### Behavioural data

Of the 22 participants with neural data, behavioural logs were available for *N* = 20; two files were overwritten due to a file-naming mistake. An early timing bug misaligned reaction time timestamps with stimulus onset; therefore, reaction time analyses for all correct trials were restricted to sessions recorded after the fix (*N* = 11). Accuracy was computed for all participants with behavioural data (*N* = 20).

**Fig. 6:**
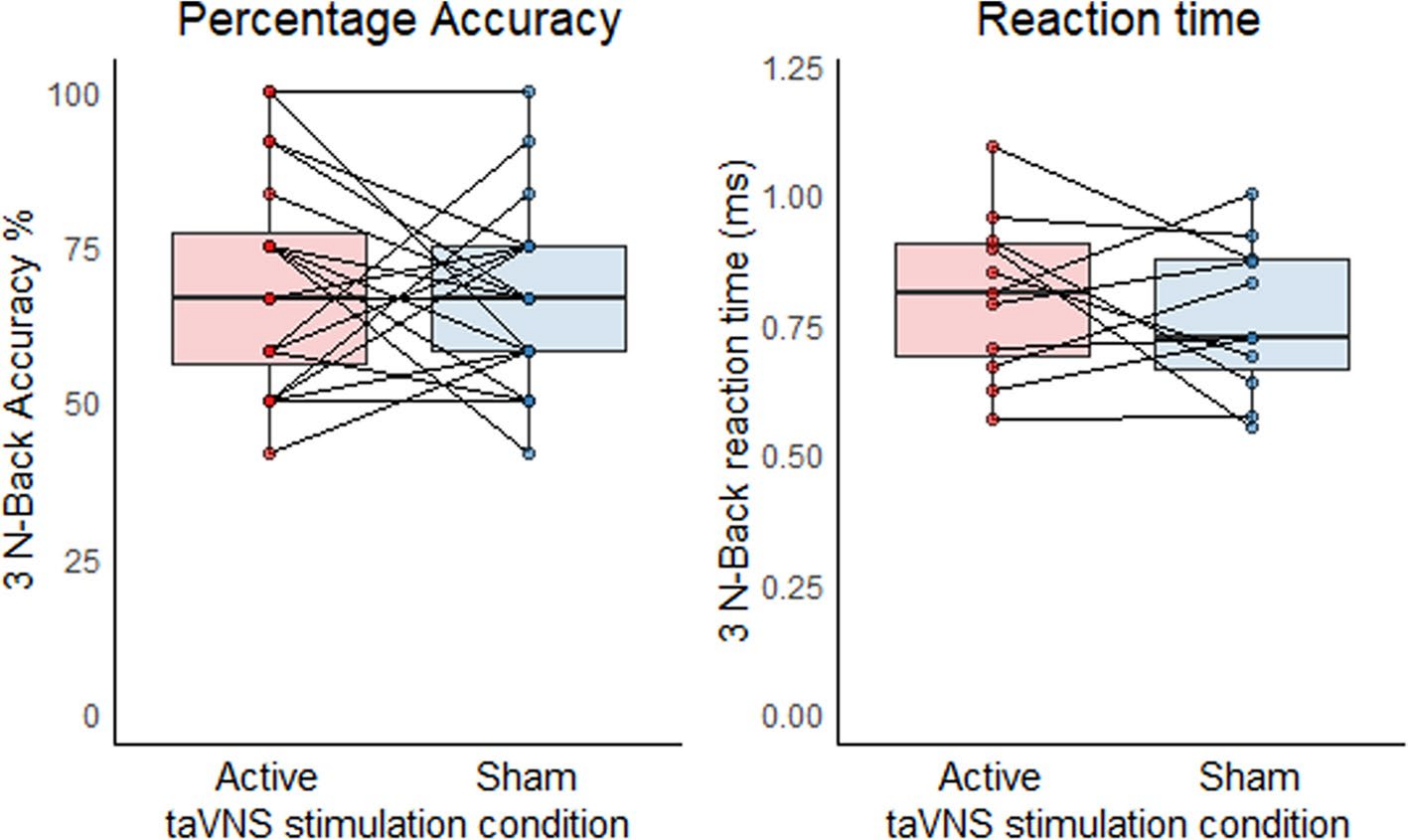
Boxplots showing the behavioural performance on the 3-back task between sham and active conditions. Box denotes first and third quartiles in the interquartile range (IQR) with median depicted as the horizontal line within. Whiskers denote range of values within 1.5 * IQR. Points denote individual participant performance, with lines connecting between performance for each condition. Left plot shows % accuracy for identifying trials with a value from 3 trials before, right plot shows reaction time for trials correctly identified as positive

Participants exhibited similar levels of accuracy in responses across active (M = 69.2%, SD = 17.5%) and sham conditions (M = 66.7%, SD = 14.6%). Similarly, reaction time was nearly equivalent across stimulation conditions (active M = 0.81 s, SD = 0.15 s; sham M = 0.76 s, SD = 0.14 s). Paired comparisons showed no significant differences between sham and active conditions for accuracy (*t*(0.53) = 0.53, *p* = .60, *d* = 0.12) or reaction time (*t*(10) = 0.79, *p* = .45, *d* = 0.24; Fig. 6). These behavioural findings should be interpreted cautiously given the fixed-order design and the reduced sample available for reaction time analysis. Sensitivity analyses indicated that the final samples had 80% power to detect within-subject effects of approximately dz = 0.66 for accuracy (*N* = 20) and dz = 0.94 for reaction time (*N* = 11), meaning that smaller behavioural effects may have gone undetected.

Exploratory analysis did not reveal a significant association between active-minus-sham changes in task-evoked ΔHbO within the right dorsolateral middle frontal gyrus and active-minus-sham changes in 3-back accuracy (Spearman’s ρ = -0.21, *p* = .37). Given the limited behavioural sample size, this analysis was considered exploratory.

### Group-average maps

**Fig. 7:**
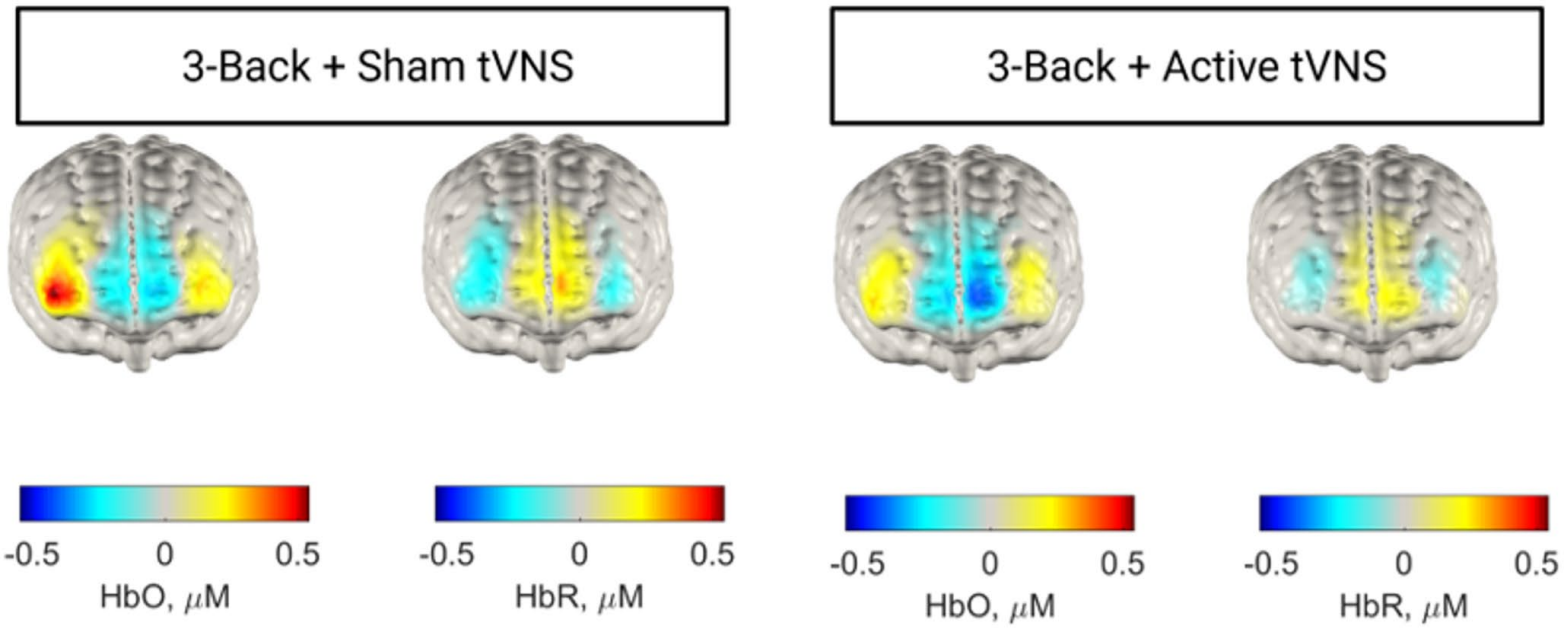
Group-level reconstruction of oxygenated haemoglobin (HbO) and deoxygenated haemoglobin (HbR) concentrations during the 3-Back task projected onto the MNI cortical surface. The task window visualised is 10–20 s post-onset, capturing the peak of activation

Group-level HbO and HbR activation maps were projected onto a MNI cortical surface to visualise the task-related activity in both conditions (Fig. 7). These show a consistent distribution of medial frontal deactivation and more lateral frontal activation in both the sham and active stimulation conditions.

### Statistical analysis of tVNS on cortical activation

**Fig. 8:**
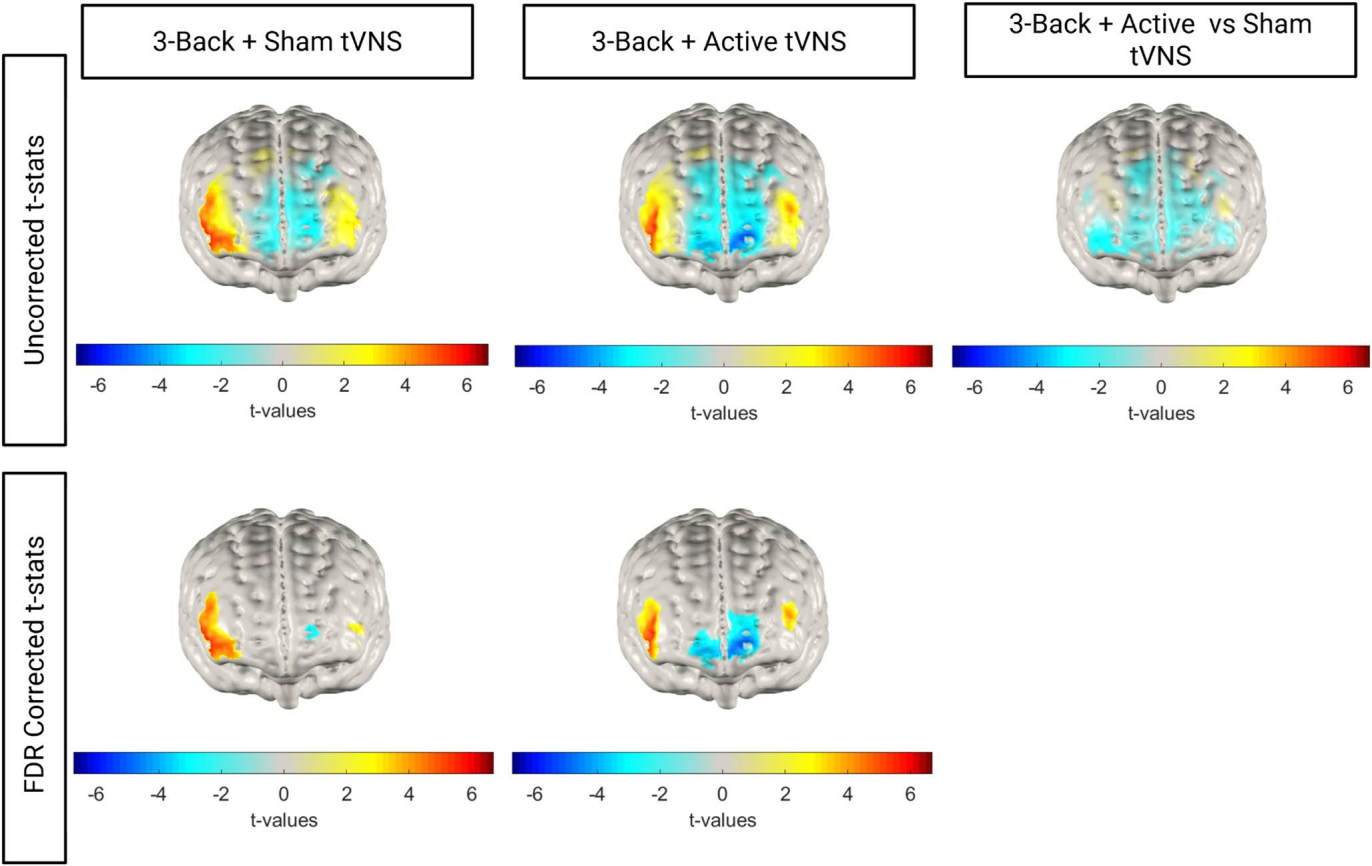
Cortical surface reconstruction of t-statistic maps comparing the block-averaged HbO concentration at baseline (− 2 to 0 s) vs. task-peak (10–20 s) at the node-level (uncorrected for multiple comparisons). No nodes survived FDR correction for the between-condition analysis

To investigate the effect of tVNS on cortical activation during the 3-back task, we conducted t-tests within each task to compare node-level changes in the task versus baseline periods for both the sham and active conditions. Uncorrected t-statistic maps are shown in Fig. 8. These demonstrate similar patterns of activation seen in the group-level HbO maps in Fig. 7.

### Parcellation analysis

Nodes were parcellated into anatomical brain regions in the sensitive areas of the AAL2 atlas and the average HbO concentration in each brain region parcel was calculated. Parcels with significant changes in HbO at the uncorrected level are shown in Table 3.

After FDR-correction, the 3-back task under sham stimulation was associated with significant increases in average parcel HbO in the left and right dorsolateral middle frontal gyrus, the left inferior frontal gyrus (triangular part), the left inferior frontal gyrus (orbital part) and the right lateral orbitofrontal cortex. Significant reductions in HbO after FDR-correction were seen in the left and right superior medial frontal gyri and medial orbital frontal cortices. For the 3-back task under active tVNS, after FDR-correction, the same regions had significant increases and decreases in HbO except the left dorsolateral middle frontal gyrus which significantly increased during sham stimulation but not active stimulation.

**Fig. 9:**
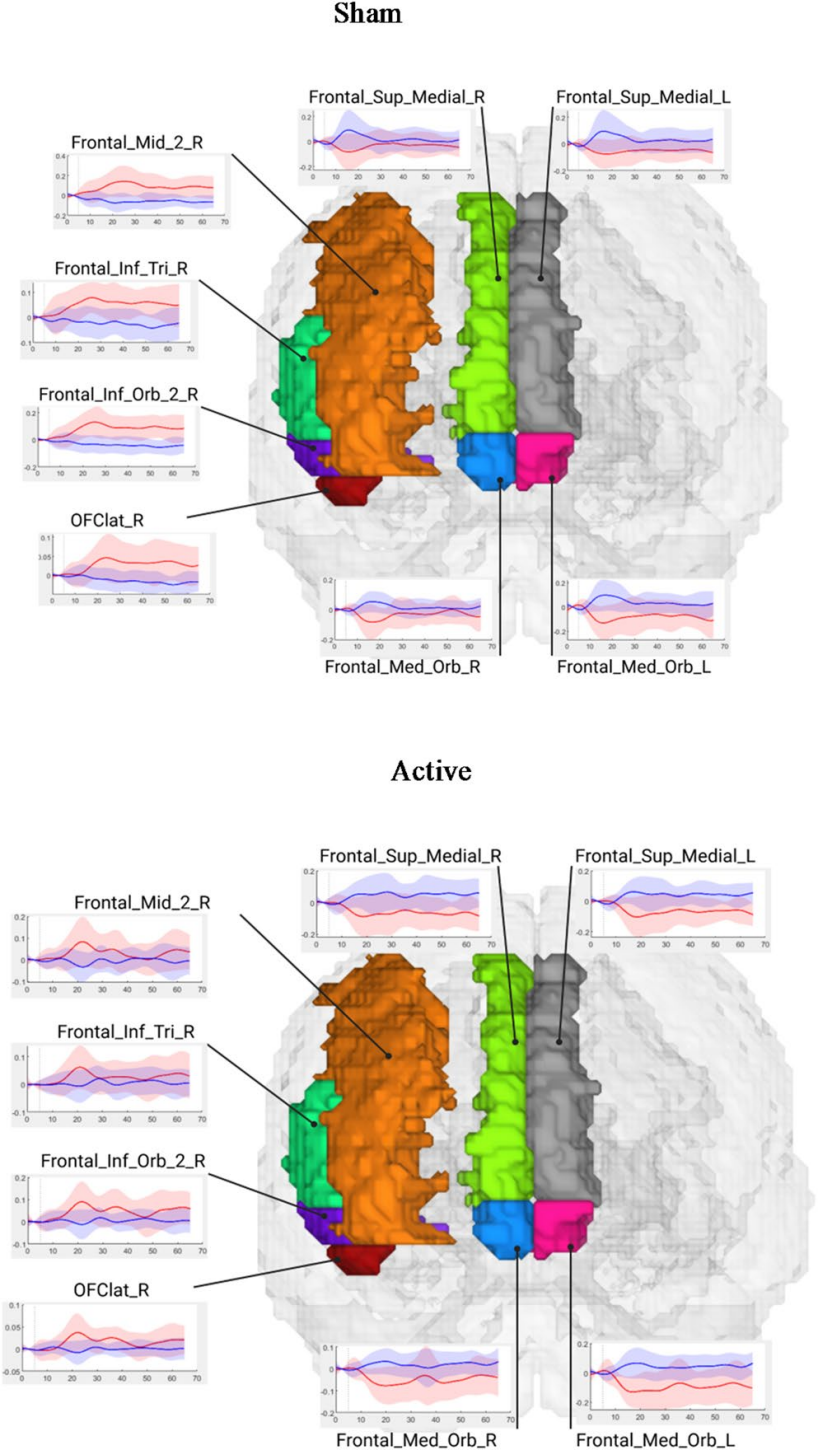
Group-level block-averaged haemodynamic response functions for statistically significant AAL2 parcels. Mean (solid line) and SD (shaded) of HbO (red) and HbR (Blue) concentrations. The dotted line indicates stimulus onset

There were no significant differences between parcel-wide HbO changes between the active and sham conditions (Supplementary Material, Table 1). Because sham stimulation always preceded active stimulation, these between-condition comparisons should be interpreted as exploratory and potentially confounded by order-related fatigue or habituation. Group-level block-averaged haemodynamic response functions for parcels showing significant task-related effects are shown in Fig. 9.

**Table 3:** Parcel-wise changes in oxygenated haemoglobin (HbO) during the 3-back task relative to baseline

	Parcels	N	Baseline HbO (µM)		Task HbO (µM)		Δ HbO (µM)	*p*	FDR *p*	Cohen’s *d*
			*M*	*SD*	*M*	*SD*				
Sham tVNS	Frontal_Sup_2_L	22	0.012	0.026	− 0.023	0.116	− 0.034	0.198	0.228	− 0.28
	Frontal_Sup_2_R	22	0.011	0.029	0.006	0.107	− 0.005	0.822	0.881	− 0.05
	Frontal_Mid_2_L	21	0.004	0.013	0.055	0.104	0.051	0.032	0.053	0.50
	Frontal_Mid_2_R	21	0.010	0.022	0.115	0.131	0.106	0.001	**0.003**	0.85
	Frontal_Inf_Tri_L	16	0.000	0.013	0.034	0.064	0.035	0.054	0.074	0.52
	Frontal_Inf_Tri_R	19	0.003	0.012	0.057	0.063	0.054	0.002	**0.005**	0.86
	Frontal_Inf_Orb_2_R	22	0.003	0.013	0.097	0.086	0.094	< 0.001	**0.001**	1.05
	Frontal_Sup_Medial_L	22	0.004	0.023	− 0.071	0.105	− 0.074	0.004	**0.011**	− 0.68
	Frontal_Sup_Medial_R	22	0.004	0.020	− 0.065	0.128	− 0.069	0.022	**0.041**	− 0.53
	Frontal_Med_Orb_L	22	0.014	0.021	− 0.112	0.132	− 0.126	< 0.001	**0.002**	− 0.92
	Frontal_Med_Orb_R	22	0.005	0.019	− 0.072	0.084	− 0.077	< 0.001	**0.002**	− 0.95
	OFCant_L	22	0.000	0.011	0.001	0.053	0.001	0.939	0.939	0.02
	OFCant_R	22	0.003	0.014	0.032	0.095	0.029	0.162	0.203	0.31
	OFClat_L	22	− 0.004	0.013	0.041	0.102	0.045	0.042	0.063	0.46
	OFClat_R	18	0.002	0.006	0.031	0.044	0.029	0.015	**0.032**	0.64
Active tVNS	Frontal_Sup_2_L	22	0.001	0.016	− 0.055	0.112	− 0.057	0.035	0.058	− 0.48
	Frontal_Sup_2_R	22	0.000	0.020	− 0.029	0.110	− 0.029	0.233	0.269	− 0.26
	Frontal_Mid_2_L	21	− 0.003	0.015	0.045	0.100	0.047	0.042	0.063	0.47
	Frontal_Mid_2_R	21	0.000	0.017	0.061	0.094	0.061	0.006	**0.012**	0.68
	Frontal_Inf_Tri_L	16	− 0.008	0.011	0.021	0.063	0.029	0.063	0.086	0.50
	Frontal_Inf_Tri_R	19	− 0.001	0.014	0.052	0.069	0.053	0.004	**0.009**	0.77
	Frontal_Inf_Orb_2_R	22	− 0.003	0.020	0.071	0.072	0.075	< 0.001	**< 0.001**	1.06
	Frontal_Sup_Medial_L	22	0.001	0.014	− 0.095	0.126	− 0.096	0.002	**0.007**	− 0.74
	Frontal_Sup_Medial_R	22	− 0.001	0.019	− 0.081	0.123	− 0.080	0.009	**0.017**	− 0.62
	Frontal_Med_Orb_L	22	0.009	0.034	− 0.125	0.110	− 0.134	< 0.001	**< 0.001**	− 1.12
	Frontal_Med_Orb_R	22	0.002	0.018	− 0.073	0.076	− 0.075	< 0.001	**0.001**	− 0.95
	OFCant_L	22	− 0.001	0.014	− 0.003	0.052	− 0.002	0.857	0.857	− 0.04
	OFCant_R	22	− 0.002	0.014	0.004	0.062	0.006	0.627	0.671	0.11
	OFClat_L	22	− 0.010	0.019	0.019	0.079	0.029	0.071	0.088	0.41
	OFClat_R	18	− 0.001	0.008	0.028	0.035	0.029	0.002	**0.007**	0.85

Baseline window = -2 to 0 s pre-task; task window = 10–20 s post-onset. ΔHbO represents task minus baseline concentration change. p-values reflect paired comparisons within each stimulation condition. FDR p-values are corrected for multiple comparisons using the false discovery rate method. Bold FDR *p* values denote significant parcels. Cohen’s d denotes within-condition effect size

Frontal_Sup_2 (left/right) – superior frontal gyrus (dorsolateral); Frontal_Mid_2 (left/right) – middle frontal gyrus (dorsolateral); Frontal_Inf_Tri (left/right) – inferior frontal gyrus, triangular part; Frontal_Inf_Orb_2_R – inferior frontal gyrus, orbital part (right); Frontal_Sup_Medial (left/right) – superior medial frontal gyrus; Frontal_Med_Orb (left/right) – medial orbital frontal cortex; OFCant (left/right) – anterior orbitofrontal cortex; and OFClat (left/right) – lateral orbitofrontal cortex

## Discussion

This is, to our knowledge, the first study to combine HD-fNIRS and tVNS during a working memory task. Simultaneous delivery of tVNS whilst recording cerebral blood flow using HD-fNIRS was feasible and well tolerated. HD-fNIRS was able to detect task-related activation in lateral frontal regions and deactivation in the medial frontal regions. No significant active-versus-sham differences in working memory performance or cerebral blood flow were observed, although these findings should be interpreted cautiously given the fixed-order design.

### Previous studies of neuroimaging during the N-Back task

Meta-analyses of dozens of fMRI studies reveal a reliable pattern of BOLD signal increases in the dorsolateral and ventrolateral prefrontal cortices, anterior cingulate, and inferior parietal lobule as task difficulty rises from 1-back to 3-back (Owen et al. 2005; Rottschy et al. 2011). A recent fMRI study of the 3-back task in healthy participants has also shown deactivation in the superior medial frontal gyrus, a key part of the default mode network that is known to become suppressed during externally focused tasks (Ashtiani and Daliri 2023).

Findings from optical imaging studies on the N-back task also show a consistent pattern (Fishburn et al. 2014). Previous low-density fNIRS studies show lateral prefrontal cortex activation from N-back tasks in healthy adults (Herff et al. 2014; Zhu et al. 2024) and have also been used to identify reduced activation in clinical populations (Chen et al. 2024). Studies using high-density fNIRS remain limited. One exception is Kothe et al. (2024) who used a 3,000-channel continuous-wave system and observed graded activation in the dorsolateral and prefrontal cortices. Our finding of increased activity in the middle frontal and inferior frontal gyri and reductions in medial superior frontal gyrus activity during the 3-back task are consistent with previous literature and provide evidence that the current HD-fNIRS paradigm reliably detects N-back task-related activation.

### Previous studies of tVNS on cognitive performance

A recent meta-analysis of 19 studies reported significant effects of auricular tVNS stimulation on executive function and measures of accuracy (Ridgewell et al. 2021). Although none of the studies used the N-back task, it included studies which share those characteristics with the N-back, namely the Flanker task, the Stroop task, and the stop-change paradigm. Improvements in performance on these tasks included faster response times, and/or higher accuracy, compared to sham stimulation control groups (Fischer et al. 2018; Ridgewell et al. 2021; Tian et al. 2023; Konjusha et al. 2023). In addition, Sun et al. (2017) reported improved working memory performance in the form of reduced errors on an executive-reaction time test. However, these studies used upwards of 15 min of stimulation and stimulated at the cymba conchae rather than the tragus.

In contrast, numerous studies have shown no significant differences in the same metrics. One such study used 4 cognitive tasks (Flanker, Stroop, Number-Letter task, and a card-sorting task) and only found increased performance on the card-sorting task, in the form of reduced switch costs (faster reaction time when rules switch) (Borges et al. 2020). Findings of cognitive effects of tVNS are inconsistent and seem to be influenced by stimulation parameters. Protocols vary in stimulation site (tragus vs. cymba conchae), polarity, frequency (often ~ 20–30 Hz), pulse width (~ 200–300 µs), intensity scaling (absolute mA vs. threshold-relative), duty cycle (continuous vs. bursts), timing (online during a task vs. offline beforehand), and dose (single session vs. multi-week courses). Several studies reporting cognitive effects have used longer stimulation periods and/or cymba conchae stimulation, whereas the present study used continuous 25 Hz left tragus stimulation applied during the task. This montage was selected as a safe, commonly used approach for feasibility testing, but may not maximise afferent vagal engagement compared with cymba conchae stimulation. An optimal cognitive tVNS protocol has not yet been established, and parameter differences may partly explain the absence of observed active-versus-sham effects in the present study.

### Relevant studies of tVNS and neuroimaging

Auricular tVNS projects to the NTS. Pathways from the NTS project to multiple neuromodulatory centres including the LC (noradrenaline), dorsal raphe (serotonin), parabrachial complex, and basal forebrain (acetylcholine). Through these pathways, tVNS could modulate cortical processing indirectly by altering arousal, sustaining vigilance, and adjusting neural gain – the sensitivity of neurons to incoming signals. The LC-noradrenaline system, in particular, has been associated with changes in attentional control, and task engagement (Chen et al. 2023).

The medial frontal deactivation observed during the 3-back task is consistent with suppression of default mode network regions during externally focused cognitive demand. However, the present study cannot determine whether continuous 25 Hz left tragus stimulation was sufficient to enhance LC-noradrenaline-mediated switching between default mode and executive-control networks. The absence of direct physiological markers of LC engagement, such as pupillometry or autonomic monitoring, limits mechanistic interpretation of the null active-versus-sham findings.

In a site-comparison fMRI study, cymba conchae stimulation produced significantly greater NTS/LC activation than earlobe sham, whereas left tragus produced a smaller, non-significant difference in the same participants; in other words, both sites trended in the expected direction, but the cymba effect was reliably larger (Yakunina et al. 2016). At the cortical level, task-free stimulation at rest has been associated with frontal decreases (Yakunina et al. 2018), while other protocols report prefrontal increases in the BOLD signal (Badran et al. 2017) and increased amplitude of low-frequency fluctuations, a marker of increased connectivity, in the left middle frontal gyrus (Zhang et al. 2022). In low density fNIRS studies, low performing healthy older adults had improvements in working memory performance and dorsomedial-ventromedial functional connectivity; this may have clinical implications for cohorts with cognitive impairment (An et al. 2025). No previous studies have concurrently delivered tVNS during a working memory task using HD-fNIRS for comparison.

### Clinical and research implications

There is a potential dual role of HD-fNIRS in clinical studies of neurological disorders with cognitive impairment - monitoring for response to treatment and monitoring disease progression. Clinical studies of non-invasive brain stimulation in cognitive disorders require accessible and scalable biomarkers (Burger et al. 2020). Demonstration of target engagement with an objective, consistent marker of cortical activity has several benefits. First, it can demonstrate proof-of-principle that a new treatment modality is having a biological effect. Second, it can potentially be used to stratify potential treatment responders and non-responders. Third, it could be used to titrate stimulation parameters to biomarker response. Fourth, early observed biomarkers such as a change in cortical activation could serve as a surrogate outcome measure in clinical trials for more efficient trial design.

In mild cognitive impairment, progression of clinical symptoms is relatively slow and requires serial assessment and longitudinal follow-up which can be time-consuming and costly (Petersen et al. 2014). Structural MRI changes such as cortical atrophy and changes in serum biomarkers e.g. plasma phosphorylated tau 217 similarly progress over a timescale of years (Jack et al. 2018). Practice effects in clinical inventories of cognitive function can confound the effects of a pharmacological or neuromodulatory intervention (Bartels et al. 2010). HD-fNIRS can provide a complementary readout of neural activity that can be performed at the bedside or in patients’ homes (Butters et al. 2025) that could serve as a biomarker of clinical interventions in MCI.

### Limitations

There are several limitations to the current study. First, as participants did not have a structural MRI, there was no subject-specific registration of the HD-fNIRS to individual anatomy and the modeling of the path of near-infrared light was performed in MNI common space. This may mean that there is slight variation in the location of specific brain regions between participants. The impact of this is minimised by the within-subject single session design and the use of a small number of anatomical parcels. Second, the cohort consisted largely of master’s students whose performance in a working memory task may not be reflective of the wider population. Third, the sham tVNS preceded active tVNS in all participants creating a potential order effect. This fixed sequence was chosen to reduce the risk that prolonged effects of active tVNS could carry over into the sham condition, as previous fMRI studies have shown sustained changes in cortical activation following tVNS (Yakunina et al. 2018). However, this design means that active stimulation was also systematically later in the session and may therefore have been more affected by cognitive fatigue, habituation, or reduced task engagement. As a result, the absence of significant active-versus-sham differences should not be interpreted as definitive evidence for the absence of an acute tVNS effect. Future studies should use randomised, counterbalanced designs, ideally with sufficient washout between stimulation conditions to minimise both carryover and sequence effects. Fourth, the statistical approach taken was of a comparison of the average HbO during the peak of activity compared to baseline; this may be less sensitive than general linear model approaches taken in fMRI that can model the time course of the haemodynamic response (Pinti et al. 2019). Fifth, although short-channel regression was used to reduce superficial haemodynamic contamination, concurrent systemic physiological measures such as heart rate, heart rate variability, respiration, or blood pressure were not recorded. Because tVNS can influence autonomic tone, changes in vascular tone or other systemic physiological signals could contribute to global haemodynamic changes independently of local neurovascular coupling. Future studies combining tVNS and HD-fNIRS should include physiological monitoring to help distinguish cortical task-related responses from systemic autonomic effects.

### Conclusions

HD-fNIRS can detect cortical activation associated with a working memory task during concurrent tVNS. In this fixed-order pilot study, no significant active-versus-sham differences were observed, but these findings should be interpreted cautiously because order-related fatigue or habituation may have influenced the active condition. Future studies should utilise randomised, counterbalanced whole-brain HD-fNIRS designs in MCI with sufficient washout to determine (a) whether HD-fNIRS can reliably differentiate between impaired and normal working memory performance and (b) whether tVNS can acutely enhance cortical blood flow during working memory tasks in people with cognitive impairment.

## Data Availability

The datasets generated during the current study are not publicly available due to ethical restrictions and the inclusion of sensitive participant data, but are available from the corresponding author on reasonable request.
